# Construction and Evaluation of a High-Quality Reference Panel for Dairy Goats

**DOI:** 10.3390/biology15141135

**Published:** 2026-07-12

**Authors:** Jianqing Zhao, Wei Wang, Jiayidaer Kamalibieke, Yuanpan Mu, Chenbo Shi, Jun Luo

**Affiliations:** 1 College of Animal Science, Xinjiang Agricultural University, Urumqi 830052, China; zhao2021@nwafu.edu.cn; 2 Shaanxi Key Laboratory of Molecular Biology for Agriculture, College of Animal Science and Technology, Northwest A&F University, Yangling 712100, China; wangweixn007@nwafu.edu.cn (W.W.); 2856943768@nwafu.edu.cn (J.K.); 2022055443@nwafu.edu.cn (Y.M.); shichenbo@nwafu.edu.cn (C.S.)

**Keywords:** dairy goat, reference panel, genotype imputation, low-coverage whole-genome sequencing

## Abstract

High-quality reference panels are important resources for genotype imputation and genomic selection in dairy goats; however, dairy-goat reference panels remain limited in sample size, population representation, and standardized workflow evaluation. In this study, 1092 dairy-goat samples from multiple populations, and public resequencing datasets, were integrated to construct and evaluate a dairy-goat reference panel for low-coverage whole-genome sequencing (lcWGS) and SNP-array data. Phasing and imputation strategies were compared using Beagle 5.4, SHAPEIT5, GLIMPSE2, and a BaseVar + Beagle pipeline, and the effects of reference-panel diversity, panel size, sequencing depth, and genotyping platform were evaluated using concordance, imputation quality score (IQS), and squared dosage correlation (r^2^). Beagle 5.4 phasing combined with GLIMPSE2 imputation reduced computational time by approximately 40% while maintaining high imputation accuracy. The largest evaluated panel (n = 1000) achieved concordance = 0.98, IQS = 0.94, and r^2^ = 0.91, while a practical population-size threshold of 600–800 individuals balanced accuracy gains and resource costs. Reference panels containing two to three genetically similar dairy-goat populations achieved stable performance, with concordance values above 0.90 and chromosome-level r^2^ values above 0.91. Low-coverage sequencing at 0.5× or above effectively reduced the loss of accuracy for low-frequency variants, whereas imputed SNP-array data increased marker density from 18 to 180 to 1850–18,500 SNPs per 1 Mb window in representative regions, corresponding to an approximately 103-fold increase, and produced more concentrated GWAS signal peaks on Chr5, Chr8, Chr17, and Chr19. These findings establish a technical framework and reference resource for genotype imputation, association analysis, and genomic selection in dairy goats.

## Simple Summary

Genotype imputation is essential for converting low-coverage sequencing and SNP-array data into dense genomic information for dairy-goat breeding. This study constructs and evaluates a high-quality reference panel for dairy goats, compares imputation tools and pipelines, and assesses the influence of reference-panel composition, population size, sequencing depth, and genotyping platform. The results provide a practical framework for improving genomic data quality and supporting more accurate genomic selection in dairy goats.

## 1. Introduction

Dairy goats occupy an important position in modern animal husbandry because of their strong environmental adaptability and favorable milk-production performance. To further improve production efficiency and optimize milk quality, a deeper understanding of the genetic mechanisms underlying economically important traits in dairy goats has become a key requirement for industrial development. High-quality reference panels, as essential tools in genomic research, integrate genetic information from large numbers of individuals and provide a critical data foundation for accurately identifying loci associated with economic traits, including milk yield, milk composition, and reproductive performance [1]. Reference-panel-based genomic prediction can improve the accuracy of breeding selection and thereby accelerate genetic improvement [2,3]. From a bioinformatic perspective, accurate phasing and genotype imputation are also essential steps in livestock genomic studies, because they determine haplotype reconstruction, genotype dosage estimation, downstream GWAS resolution, and the reliability of genomic selection when genotypes are generated from lcWGS or sparse SNP arrays.

Low-coverage whole-genome sequencing (lcWGS) and SNP-array genotyping are commonly used to obtain genetic information in dairy-goat studies. lcWGS can capture genome-wide variation at a relatively low cost, but its shallow sequencing depth often results in missing or uncertain genotypes [4]. SNP arrays can efficiently assay predefined markers; however, marker coverage is constrained by array design [5]. The effectiveness of both approaches depends strongly on the quality of the reference panel. Previous studies have shown that reference-panel size and genetic diversity directly affect imputation accuracy. In cattle, multi-breed panels, such as those generated through the 1000 Bull Genomes Project, improved the imputation of low-density genotypes to the whole-genome sequence level by integrating data across breeds [6,7]. Similarly, in sheep, within-breed reference panels can achieve imputation accuracies exceeding 80% because of higher genetic relatedness between target and reference animals [8,9]. In dairy goats, Talouarn used a French dairy-goat reference panel for sequence imputation and found that breed-specific panels yielded higher genotype concordance (0.86) than across-breed panels, although the high proportion of low-frequency variants still limited the power of association analyses. In addition, functionally prioritized variant-selection strategies, such as prioritization based on genome-wide association study (GWAS) signals, can further improve the accuracy of genomic prediction [2], providing methodological support for optimizing dairy-goat reference panels. The pipelines compared in this study differ fundamentally in their input data requirements and computational assumptions. Beagle 5.4 and SHAPEIT5 were mainly used for haplotype phasing based on called genotypes, whereas GLIMPSE2 performs lcWGS imputation by directly using genotype likelihoods together with reference haplotypes. In contrast, the BaseVar + Beagle strategy first detects variants from low-depth sequencing data and then refines and imputes genotypes using Beagle, thereby integrating variant detection and genotype imputation into a combined workflow.

Despite this progress, the construction of dairy-goat reference panels still faces several challenges: insufficient sample size limits genetic representativeness [10], limited genotyping precision can introduce data errors [11], and the lack of standardized designs hinders comparability among studies [12]. These limitations restrict the imputation performance of both lcWGS and array data and ultimately affect the reliability of genomic selection [13]. Therefore, the development of a high-quality dairy-goat reference panel, together with an efficient and accurate phasing/imputation workflow, is both practically important and urgently needed. In the present study, advanced sequencing technologies and bioinformatics approaches were used to integrate representative dairy-goat samples and construct a comprehensive, accurate, and standardized high-quality reference panel. In addition, different phasing/imputation strategies and variant-detection/imputation pipelines were systematically compared to identify an efficient workflow for lcWGS and SNP-array-based genomic studies in dairy goats. This panel is expected to provide a robust tool for dairy-goat genetic research, improve genotype imputation for lcWGS and SNP-array data, promote the wider application of these technologies in dairy-goat genomics, and contribute to the sustainable development of the dairy-goat industry.

## 2. Materials and Methods

### 2.1. Sample Collection

A total of 1092 dairy-goat samples from four breeds/populations and three geographic regions were used in this study, including Xinong Saanen dairy goats (n = 219) and Alpine dairy goats (n = 22) collected from Shaanxi Province, and Saanen dairy goats collected from the Inner Mongolia Autonomous Region (n = 543). For each goat, 5 mL of blood was collected from the jugular vein into an anticoagulant tube, stored at −80 °C, and subjected to DNA extraction using the standard phenol–chloroform method. After DNA concentration was determined, qualified samples were submitted to BGI Genomics Co., Ltd. (Shenzhen, China). for whole-genome sequencing. The average insert size was 500 bp, and the average read length was 150 bp for each individual. In addition, publicly available dairy-goat resequencing datasets were included to improve the representativeness and reproducibility of the reference panel. These datasets comprised Xinong Saanen dairy goats collected from Shaanxi Province (n = 114), Guanzhong dairy goats collected from Shaanxi Province (n = 10), and Saanen dairy goats collected from Zhejiang Province (n = 184). The public data were obtained from the Genome Sequence Archive in the National Genomics Data Center, China National Center for Bioinformation/Beijing Institute of Genomics, Chinese Academy of Sciences, under accession number CRA017705, available at https://ngdc.cncb.ac.cn/gsa (accessed on 6 February 2025), and from the NCBI Sequence Read Archive under BioProject accession number PRJNA1127047, available at https://www.ncbi.nlm.nih.gov/sra (accessed on 31 July 2025). The key characteristics of the datasets used for reference-panel construction are summarized in Table 1.

### 2.2. Sequencing and Variant Detection

Raw paired-end sequencing reads were first filtered using Trimmomatic v0.39 to remove adaptor sequences and low-quality reads. The clean reads were then aligned to the goat reference genome ARS1.2 (GCF_001704415.2) using BWA-MEM v0.7.15-r1140. SAMtools v1.17 was used to generate and index BAM files, and the BAM files were subsequently sorted. Potential duplicate reads were removed using Picard. Variant calling was performed using the GATK package v3.8-1-0-gf15c1c3ef. Specifically, the “HaplotypeCaller”, “GenotypeGVCFs”, and “SelectVariants” modules were used to generate and extract SNPs. The initially detected SNPs were further filtered using the GATK “VariantFiltration” module with the following parameters: QD < 2.0, FS > 60.0, MQ < 40.0, MQRankSum < −12.5, ReadPosRankSum < −8.0, and SOR > 3.0. In addition, variants were filtered according to the average sequencing-depth criterion of <1/3× or >3× across all individuals. After quality control, high-confidence SNPs were retained for subsequent reference-panel construction and genotype-imputation analyses. Functional annotation of the retained SNPs was performed using ANNOVAR version 2020Apr28.

### 2.3. Construction of a High-Quality Reference Panel

The high-quality reference panel was constructed and evaluated from three perspectives. First, multi-breed and single-breed dairy-goat reference panels were generated to evaluate the effect of dairy-goat breed diversity on imputation performance. Specifically, the single-breed panel was constructed using Saanen dairy-goats, whereas the multi-breed dairy-goat panels were constructed by combining the dairy-goat breeds/populations included in this study, including Xinong Saanen, Alpine, Guanzhong, and Saanen dairy-goat populations from Shaanxi Province, Zhejiang Province, and the Inner Mongolia Autonomous Region. Second, reference panels with different population sizes (400, 600, 800, and 1000 individuals) were constructed to assess the effect of reference-panel size. Third, reference panels consisting of dairy-goat populations alone and mixed panels containing dairy-goat populations together with publicly available non-dairy goat populations were established to compare the effect of broader breed diversity on imputation accuracy. The exact breed/population composition of each panel was kept consistent with the grouping design used in the reference-panel diversity analysis. Based on the results of these evaluations, the newly sequenced and downloaded datasets were integrated to generate a final high-quality dairy-goat reference panel for subsequent genotype imputation.

The grouping strategy for reference-panel construction was supported by previously published whole-genome resequencing analyses based on overlapping dairy-goat datasets, in which phylogenetic analysis, principal component analysis (PCA), ADMIXTURE analysis, and linkage-disequilibrium/genetic-relatedness analyses showed that Saanen-related and other dairy-goat populations clustered more closely with each other than with non-dairy-goat populations [14,15]. Therefore, in the present study, we used these published population-structure results as genetic evidence for grouping closely related dairy-goat populations, while focusing the current analysis on the effect of reference-panel composition, population size, sequencing depth, and genotyping platform on imputation performance.

### 2.4. Evaluation of Genotype-Imputation Accuracy

To comprehensively evaluate genotype-imputation accuracy, three widely used metrics were applied: concordance rate, imputation quality score (IQS), and the squared correlation coefficient (r^2^) [16,17]. Genotype concordance was calculated using a probability-weighted method and can be described as the ratio of the sum of matching probabilities across genotype categories to the total number of genotypes [18]. This approach explicitly accounts for the probabilistic nature of imputation. The squared dosage correlation (r^2^) was calculated as the square of the Pearson correlation coefficient between observed genotypes and imputed dosages. Observed genotypes were encoded according to minor-allele counts: major-allele homozygotes were coded as 0, minor-allele homozygotes as 2, and heterozygotes (0|1 or 1|0) as 1. Dosage values were extracted from Beagle 5.4 output and ranged from 0 to 2.

As a complementary metric, IQS was used to correct for agreement expected by chance. IQS quantifies the deviation between the observed matching rate (Po) and the theoretical random matching rate (Pc), thereby providing a probability-adjusted measure of imputation accuracy. The theoretical random matching rate was calculated based on the product of genotype marginal frequencies, which approximates the probability of chance matches under random genotype assignment [19]. To represent chromosomes of different lengths, one long, one medium-length, and one short chromosome were selected for downstream imputation-accuracy assessment: Chr1, Chr13, and Chr25, respectively. These chromosomes were chosen to provide a practical representation of length-dependent differences in marker number, haplotype complexity, and imputation difficulty while maintaining feasible computational costs. We acknowledge that evaluation based on three chromosomes cannot fully capture all genome-wide variation in recombination rate, linkage-disequilibrium structure, gene density, or breed-specific haplotype diversity. Therefore, the results from Chr1, Chr13, and Chr25 were interpreted mainly as a representative benchmark for comparing different imputation strategies under the same chromosomal settings, rather than as an exhaustive whole-genome estimate. Because all reference-panel designs and imputation methods were evaluated using the same three chromosomes, potential chromosome-selection effects were expected to have a limited influence on the relative comparison among methods. Because the present study was designed as a controlled methodological evaluation rather than a replicated experimental design, the comparisons of imputation performance were interpreted descriptively. Formal standard deviations, confidence intervals, and statistical tests were not calculated because replicate-level or genomic-window-level estimates were not generated in the original workflow.

### 2.5. Evaluation of Imputation Methods

To compare the effects of different phasing and imputation methods on imputation accuracy, all methods were evaluated using the same reference panel, target datasets, selected chromosomes, accuracy metrics, and computational environment. Beagle 5.4 and SHAPEIT5 were first evaluated as alternative phasing methods using the same genotype dataset. For the comparison of imputation tools, Beagle 5.4 and GLIMPSE2 were evaluated using the same reference panel and target datasets to reduce potential bias caused by differences in input data. When phased haplotypes were required for downstream imputation, the phasing procedure was kept consistent within each comparison. Because the compared software packages have different parameter structures, tool-specific settings were configured according to the requirements of each program and were kept consistent across repeated analyses within the same software.

Running time was recorded separately for each selected chromosome rather than per sample or for the complete genome, and all runtime comparisons were performed under the same computational environment. In addition, a combined pipeline integrating the low-depth resequencing variant-detection tool BaseVar with Beagle 5.4 was compared with GLIMPSE2. Since BaseVar v0.8.0 + Beagle 5.4 includes both low-depth variant detection and genotype refinement/imputation, whereas GLIMPSE2 performs genotype imputation using genotype likelihoods and reference haplotypes, this comparison was interpreted as a workflow-level comparison rather than a pure imputation-algorithm comparison. Both pipelines were evaluated using the same low-depth target data, reference panel, selected chromosomes, and accuracy metrics to improve the comparability of the results. Based on these comparisons, the Beagle 5.4 + GLIMPSE2 strategy was selected because Beagle 5.4 provided efficient and accurate haplotype phasing for called genotypes, whereas GLIMPSE2 showed faster imputation performance for low-coverage sequencing data using genotype likelihoods and reference haplotypes. Therefore, this combined strategy balanced imputation accuracy and computational efficiency and was considered suitable for large-scale reference-panel applications.

### 2.6. Comparison of Imputation Performance for lcWGS and SNP-Array Data

To investigate imputation performance for lcWGS and SNP-array data, 10× resequencing data were down-sampled to 1×, 0.5×, and 0.1×. In parallel, 25K liquid SNP-array data generated for dairy-goat selection in this study were imputed using the high-quality reference panel described above. These analyses were used to assess the impact of sequencing depth and SNP-array data on imputation accuracy.

## 3. Results

### 3.1. Comparative Analysis of Phasing and Imputation Time and Accuracy

**Figure 1:**
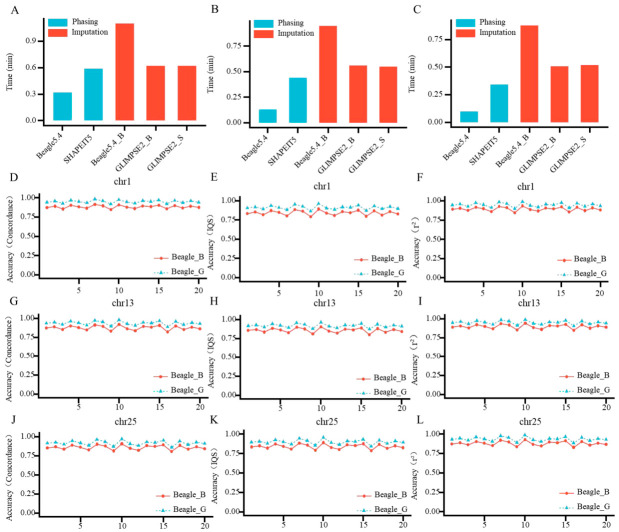
Comparison of phasing and imputation software. (**A**–**C**) Phasing and imputation time for chromosomes 1, 13, and 25. (**D**–**L**) Comparison of imputation accuracy among different imputation software tools for chromosomes 1, 13, and 25.

During the construction of a large-scale reference panel, computational efficiency is a key determinant of feasibility. A systematic performance evaluation revealed tool-dependent differences among software packages. Across Chr1, Chr13, and Chr25, Beagle 5.4 showed the shortest phasing time, followed by SHAPEIT5. Comparison of imputation speed further indicated that GLIMPSE2 was faster than Beagle 5.4 (Figure 1A–C). After phasing with Beagle 5.4, imputation accuracy was evaluated using both Beagle 5.4 and GLIMPSE2 (Figure 1D–L). Although the two imputation tools differed slightly in concordance, IQS, and r^2^, both maintained high overall accuracy across the three representative chromosomes. Therefore, a hybrid strategy in which Beagle 5.4 is used for phasing and GLIMPSE2 for efficient imputation is recommended for subsequent analyses. This strategy reduced total computational time by approximately 40% while maintaining high genotype-recall performance.

### 3.2. Effect of Different Variant-Detection Pipelines on Imputation Accuracy

**Figure 2:**
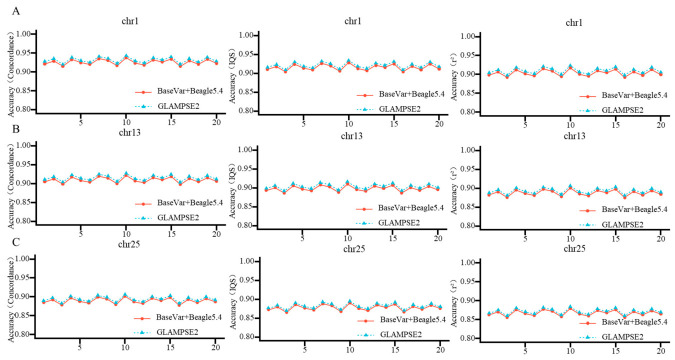
Effect of variant-detection and imputation pipelines on imputation accuracy. (**A**) Comparison of imputation accuracy between the BaseVar + Beagle 5.4 pipeline and the GLIMPSE2 pipeline on Chromosome 1. (**B**) Comparison on Chromosome 13. (**C**), Comparison on Chromosome 25.

Two genotype-detection and imputation pipelines, BaseVar + Beagle 5.4 and GLIMPSE2, were systematically evaluated on Chr1, Chr13, and Chr25 in dairy goats (Figure 2). Both methods showed strong imputation performance across the three chromosomes, with average accuracies exceeding 0.85. Chr1 displayed higher accuracy than Chr13 and Chr25, whereas the accuracy distributions for Chr13 and Chr25 were wider but remained within the same overall range. The accuracy curves of BaseVar + Beagle 5.4 and GLIMPSE2 largely overlapped across repeated experiments, indicating that the two pipelines were comparable at the genomic scale. In low-coverage regions, BaseVar + Beagle 5.4 showed a narrower fluctuation range than GLIMPSE2, suggesting that it may be more robust for sparse genotype data. The consistency between the two methods indicates that coordinated optimization of variant detection and imputation algorithms can influence final accuracy; future studies may further integrate different modules to improve overall performance.

### 3.3. Effect of Reference Panels on Imputation Accuracy

#### 3.3.1. Effect of Reference-Panel Diversity on Imputation Accuracy

**Figure 3:**
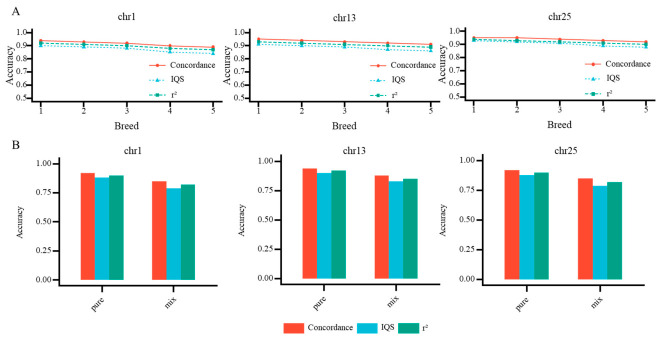
Effect of reference-panel diversity on imputation accuracy for Chr1, Chr13, and Chr25. (**A**) Comparison of reference-panel construction and imputation accuracy among different dairy-goat breeds. (**B**) Comparison of imputation accuracy between mixed panels of dairy and non-dairy goats and pure dairy-goat panels.

Multidimensional evaluation revealed the effect of reference-panel composition on dairy-goat genome imputation accuracy (Figure 3). The three accuracy metrics—concordance, IQS, and r^2^—were stable across changes in breed diversity for Chr1, Chr13, and Chr25. When the reference panel contained two to five dairy-goat breeds and the total sample size was fixed at 400 individuals, concordance values for Chr1, Chr13, and Chr25 remained above 0.90, and IQS and r^2^ showed similar ranges of variation. These results indicate that moderate breed diversity (at least two breeds) did not reduce concordance below 0.90 in the evaluated chromosomes. Of note, the single-breed Saanen dairy-goat panel achieved the highest concordance on Chr1, but r^2^ decreased on Chr13 and Chr25. This suggests that a high-density single-breed panel may improve chromosome-specific imputation efficiency while reducing accuracy in more complex genomic regions. In addition, compared with the pure dairy-goat panel, the mixed panel containing dairy and non-dairy goats showed lower IQS and r^2^ values on Chr1, Chr13, and Chr25, with a particularly evident decrease in concordance on Chr25. This finding suggests that cross-type mixed panels may introduce potential risks to chromosome-level imputation robustness. Overall, when constructing regional dairy-goat reference panels, priority should be given to two or three breeds with similar genetic backgrounds and balanced sample distributions. This strategy can maintain genome-wide imputation accuracy while minimizing bias caused by population-structure heterogeneity.

#### 3.3.2. Effect of Reference-Panel Size on Imputation Accuracy

**Figure 4:**
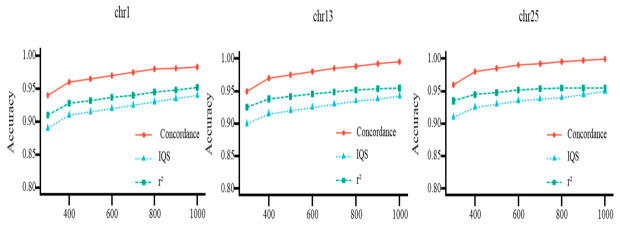
Effect of reference-panel size on imputation accuracy.

Reference-panel size showed a clear population-size effect on genotype-imputation accuracy (Figure 4). As the reference population increased to 1000 individuals, accuracy measured by concordance, IQS, and r^2^ improved stepwise. Concordance reached the highest value at the largest scale (n = 1000; 0.98), exceeding IQS (0.94) and r^2^ (0.91). However, the marginal benefit declined as sample size increased: the average increase in accuracy was 8.3% from 400 to 600 individuals but decreased to 2.1% from 800 to 1000 individuals. Methodological differences were most pronounced at low sample sizes; for example, the difference between concordance and r^2^ reached 0.15 at n = 400, but the gap gradually narrowed after the reference size exceeded 800 individuals. A practical threshold of 600–800 individuals was identified, within which accuracy gains and resource costs were optimally balanced. This provides a quantitative basis for selecting reference-panel size in practical applications.

### 3.4. Effects of Sequencing Depth and SNP-Array Data on Imputation Accuracy

**Figure 5:**
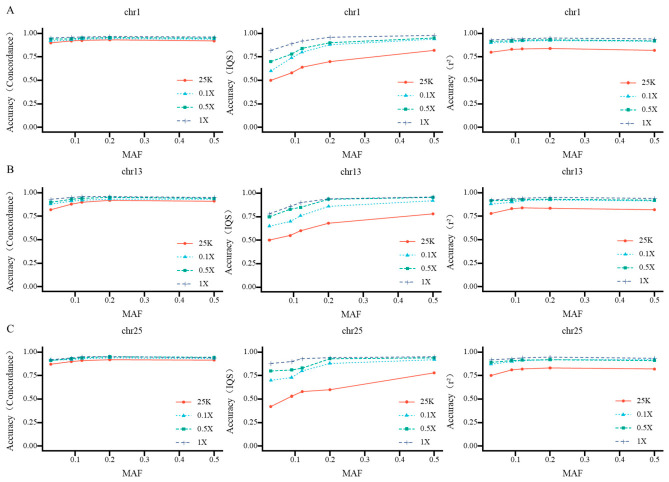
Effects of low-coverage sequencing and SNP-array platforms on imputation accuracy. (**A**) Evaluation of imputation accuracy for 25 K, 0.1×, 0.5×, and 1× sequencing depths on Chromosome 1 using concordance, IQS, and r^2^. (**B**) Evaluation on Chromosome 13. (**C**) Evaluation on Chromosome 25.

As shown in Figure 5, imputation accuracy was affected by both sequencing depth and minor-allele frequency (MAF). Across all three chromosomes (Chr1, Chr13, and Chr25), concordance, IQS, and r^2^ increased with sequencing depth from 0.1× to 1×, especially in low-MAF intervals (MAF < 0.2). However, the improvement was not linear. For example, r^2^ on Chr1 increased from 0.1× to 0.5× and then changed only slightly from 0.5× to 1×, indicating that very low coverage (<0.5×) imposes a stronger constraint on imputation accuracy. These results provide quantitative support for the practical use of lcWGS in dairy-goat genomic studies, especially when SNP-array marker density is insufficient for low-frequency variant recovery.

Chromosome-level comparison showed that Chr25 was most sensitive to sequencing depth. At MAF = 0.1, the IQS of 0.1× sequencing on Chr25 was lower than those on Chr1 and Chr13, but the differences gradually decreased when sequencing depth increased to 1×. Notably, sequencing depth had a smaller effect on high-MAF intervals (MAF ≥ 0.2). On Chr13, the concordance difference between 1× and 0.5× sequencing decreased from 0.18 at MAF = 0.1 to 0.04 at MAF = 0.5. These results indicate that a sequencing depth of at least 0.5× can reduce the loss of imputation accuracy for low-frequency variants (MAF < 0.1). To maintain high accuracy across chromosomes and for rare-variant analyses, a sequencing depth above 0.5× is recommended. Nevertheless, the overall imputation accuracy indicated that the dairy-goat reference panel generated here can be used in subsequent experimental and breeding studies. To improve the clarity of the main findings, the key imputation results are summarized in Table 2.

### 3.5. Evaluation of Imputation Performance Using the Dairy-Goat Reference Panel

**Figure 6:**
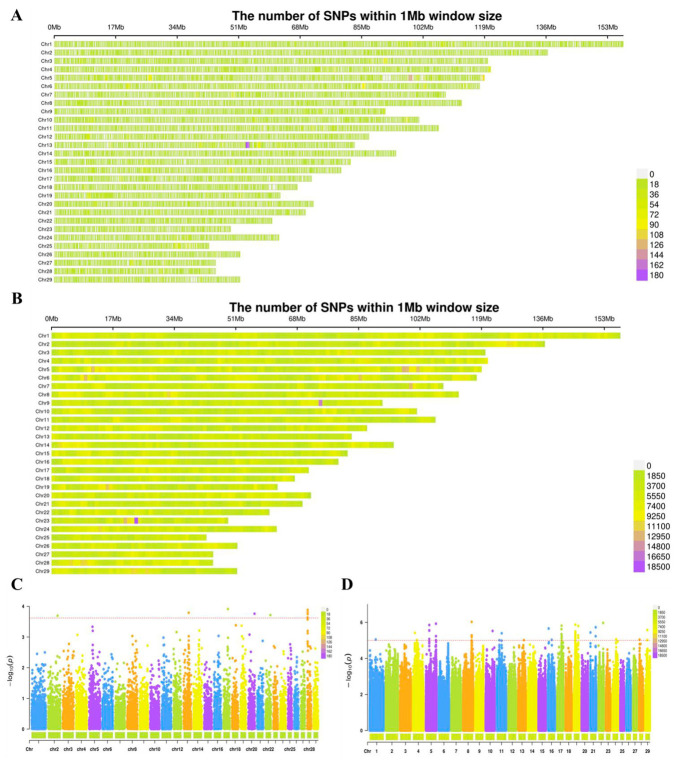
Evaluation of imputation performance using the dairy-goat reference panel. (**A**) Density plot of SNP distribution in the SNP-array data. (**B**) Density plot of SNP distribution after imputation using the reference panel. (**C**) Manhattan plot of GWAS based on SNP-array data. (**D**) Manhattan plot of GWAS after imputation using the reference panel.

Based on the newly constructed dairy-goat genomic reference panel, genotype data from the target population were systematically imputed. As shown in Figure 6, comparison of SNP-density distributions and GWAS results before and after imputation confirmed that the reference panel improved genomic data quality. The SNP-density comparison between Figure 6A (before imputation) and Figure 6B (after imputation) showed that SNP coverage density increased across chromosomal regions after imputation. In particular, within the continuous detection interval from 17 Mb to 153 Mb, the number of SNPs per 1 Mb window increased from 18 to 180 before imputation to 1850–18,500 after imputation, corresponding to an approximately 103-fold increase in representative windows. Moreover, the spatial distribution of SNPs across chromosomes became more uniform after imputation, and the distance between variant sites decreased, indicating that the reference panel increased the completeness of missing genotype regions.

The comparison of Manhattan plots further supported the imputation effect (Figure 6C versus Figure 6D). After imputation, association signals became more regionally clustered, with clear signal peaks formed in key regions such as Chr5, Chr8, Chr17, and Chr19. This focusing of association signals indicates that the reference panel not only increased SNP density from 18 to 180 to 1850–18,500 SNPs per 1 Mb window in representative regions but also supported finer regional localization of GWAS signals. These results confirm that the newly constructed reference panel provides a high-density genotype-imputation solution for dairy-goat genomic studies and establishes a data foundation for subsequent GWAS and genomic-selection analyses.

## 4. Discussion

This study systematically constructed a high-quality reference panel for dairy goats and evaluated the mechanisms and application value of genome-wide imputation from multiple perspectives. As a reference-panel construction and validation study tailored to the genomic features of dairy goats, this work evaluated algorithm selection, pipeline optimization, population-size control, and data-platform compatibility, thereby providing an important methodological basis for dairy-goat genomics research.

This study optimized phasing and imputation algorithms for dairy-goat genomic research (Figure 1). Conventional genotype imputation often uses a single tool for both phasing and imputation [20]. In contrast, the present results suggest that combining Beagle 5.4 for phasing with GLIMPSE2 for imputation can reduce computational time by approximately 40% while maintaining the high imputation accuracy shown by concordance, IQS, and r^2^ in Figure 1. This indicates that phasing places high demands on the accuracy of haplotype inference, whereas imputation depends more strongly on haplotype-matching efficiency. Differences in algorithmic architecture therefore provide a theoretical basis for separating the two steps [21]. Compared with Minimac4, which is widely used in human genomic studies, the speed advantage of GLIMPSE2 in cross-species applications may be related to its graph-based haplotype-clustering algorithm [22], which may better accommodate linkage-disequilibrium patterns characteristic of inbred populations [23]. Notably, although BaseVar + Beagle 5.4 and GLIMPSE2 showed comparable overall accuracy (Figure 2), the former was more robust in low-coverage regions, as reflected by a reduced IQS fluctuation range. This advantage may be associated with the dynamic sequencing-error correction mechanism of BaseVar [24]. Therefore, in practical applications, imputation pipelines should be selected according to data type, such as high-density SNP arrays or lcWGS, which is important for improving resource-use efficiency [25]. This result indicates that separating phasing from imputation can be useful in livestock datasets where computational efficiency and haplotype accuracy must be balanced, especially when reference panels are expanded for routine breeding applications. Large-scale human reference-panel resources have also demonstrated the value of comprehensive variant catalogs for downstream genetic analyses [26].

The present study confirmed that the size and population genetic structure of the reference panel have important effects on imputation accuracy (Figure 3 and Figure 4). From a biological perspective, the improvement in imputation accuracy with increasing reference-panel size can be explained by the more complete representation of haplotype diversity and linkage-disequilibrium structure in the target population. Genotype imputation relies on the sharing of chromosomal segments between reference and target individuals; therefore, a larger reference panel is more likely to contain haplotypes that are identical or highly similar to those carried by the target animals. This is particularly important for low-frequency variants, because rare haplotypes are less likely to be captured in small reference panels. In the present study, the marginal gain became smaller when the panel size reached approximately 600–800 individuals, suggesting that the major common haplotypes of the dairy-goat populations were largely represented at this scale, although further expansion may still benefit rare-variant imputation. Similar observations have been reported in cattle and sheep, where larger reference panels generally improved sequence-level imputation accuracy, but the benefit depended strongly on the extent of haplotype sharing between reference and target populations [6,7,8,9,11].

The effect of reference-panel composition also has an important biological basis. Reference panels composed of genetically similar dairy-goat populations provided more stable imputation performance than panels containing more divergent goat populations, indicating that genetic relatedness and shared haplotype structure are more important than simply increasing broad breed diversity. Many modern dairy-goat populations have experienced intensive artificial selection for milk-production traits and may therefore share more similar genomic backgrounds and haplotype segments than non-dairy-goat populations. This interpretation is consistent with our previously published population-genomic studies, in which phylogenetic analysis, principal component analysis, ADMIXTURE analysis, and linkage-disequilibrium analyses showed that Saanen-related and other dairy-goat populations clustered more closely with each other than with non-dairy-goat breeds [14,15]. Therefore, the stable performance of dairy-goat-oriented reference panels in the present study is biologically consistent with the closer genetic relationship among these populations. Comparable findings have also been reported in other livestock species, where within-breed or closely related multi-breed reference panels often outperform highly divergent panels when the target animals share more haplotypes with the reference population [6,7,8,9,13].

The comparison of sequencing depths and genotyping platforms (Figure 5) revealed the application value of low-coverage sequencing and reference-panel-based imputation for dairy-goat genomic studies. From a practical perspective, lcWGS combined with a dairy-goat-specific reference panel provides a cost-effective strategy for increasing marker density and recovering variants that are not directly captured by sparse SNP arrays. Compared with SNP-array data, imputed lcWGS or array-based genotypes can provide a denser and more continuous marker distribution across the genome, which may improve the apparent resolution of downstream association analyses. In the GWAS comparison, imputation increased local marker density from 18 to 180 to 1850–18,500 SNPs per 1 Mb window in representative regions and made association signals more regionally concentrated (Figure 6). This result suggests that reference-panel-based imputation can help recover informative variants and improve the detection of genomic regions associated with dairy-goat traits. However, these post-imputation association signals should be interpreted as improved association signals rather than definitive causal variants. Further functional validation, independent population verification, and integration with transcriptomic or phenotypic data will be required to confirm the biological roles of candidate loci detected after imputation. These observations are consistent with previous studies showing that increased marker density and imputation to sequence-level genotypes can enhance genomic evaluation, GWAS resolution, and candidate-locus/QTL discovery across species [27,28,29,30].

Several limitations should also be noted. First, although the reference panel integrated 1092 dairy-goat individuals from multiple breeds/populations and geographic regions, the representation of some populations, such as Alpine and Guanzhong dairy goats, remained limited. Further expansion of the reference panel with more individuals from diverse dairy-goat breeds will be useful for improving the imputation of rare variants and population-specific haplotypes. Second, the comparisons of imputation performance in this study were mainly descriptive because the original workflow did not generate replicate-level or genomic-window-level estimates required for calculating standard deviations, confidence intervals, or formal statistical tests. Future studies should incorporate repeated down-sampling or genome-window-based evaluation to quantify statistical uncertainty in imputation-performance comparisons. Third, although post-imputation GWAS showed increased marker density and clearer regional association signals, these signals require further validation before they can be interpreted as causal loci. Despite these limitations, the present study provides a useful dairy-goat reference resource and a practical imputation framework for lcWGS, SNP-array imputation, GWAS, and genomic selection in dairy goats.

## 5. Conclusions

This study constructed and evaluated a high-quality dairy-goat reference panel using 1092 individuals from multiple populations. The results showed that Beagle 5.4 phasing combined with GLIMPSE2 imputation provided an efficient and accurate strategy for genotype imputation, reducing total computational time by approximately 40%. Reference panels composed of two to three genetically similar dairy-goat breeds achieved stable performance, with concordance values above 0.90 and chromosome-level r^2^ values above 0.91, and a panel size of 600–800 individuals offered a practical balance between accuracy and cost. Low-coverage sequencing at 0.5× or above reduced the loss of imputation accuracy for low-frequency variants, while SNP-array imputation increased marker density from 18 to 180 to 1850–18,500 SNPs per 1 Mb window in representative regions and produced more concentrated GWAS signals. Overall, this reference panel provides a useful genomic resource and technical framework for genotype imputation, association analysis, and genomic selection in dairy goats.

## Figures and Tables

**Table 1:** Summary of datasets used for dairy-goat reference-panel construction.

Data Source	Breed/Population	Geographic Origin	Number of Individuals	Use in This Study
Newly sequenced data	Xinong Saanen dairy goat	Shaanxi Province	219	Reference-panel construction
Newly sequenced data	Alpine dairy goat	Shaanxi Province	22	Reference-panel construction
Newly sequenced data	Saanen dairy goat	Inner Mongolia Autonomous Region	543	Reference-panel construction
Public resequencing data	Xinong Saanen dairy goat	Shaanxi Province	114	Reference-panel construction
Public resequencing data	Guanzhong dairy goat	Shaanxi Province	10	Reference-panel construction
Public resequencing data	Saanen dairy goat	Zhejiang Province	184	Reference-panel construction
Total	Four dairy-goat breeds/populations	Three geographic regions	1092	Final reference panel

**Table 2:** Summary of the main imputation results obtained in this study.

Analysis Aspect	Comparison or Scenario	Main Result	Interpretation
Phasing/imputation strategy	Beagle 5.4 + GLIMPSE2	Reduced total computational time by approximately 40% while maintaining high imputation accuracy	Balanced accuracy and computational efficiency
Reference-panel size	n = 400, 600, 800, 1000	Largest panel, n = 1000, achieved concordance = 0.98, IQS = 0.94, and r^2^ = 0.91	Larger panels improved accuracy, but 600–800 individuals provided a practical balance
Reference-panel diversity	Single-breed, multi-breed dairy-goat, and mixed dairy/non-dairy panels	Panels containing two to three genetically similar dairy-goat populations showed stable performance, with concordance > 0.90 and chromosome-level r^2^ > 0.91	Genetically similar dairy-goat populations improved reference-panel performance
Sequencing depth	1×, 0.5×, 0.1× lcWGS	0.5× or above reduced the loss of accuracy for low-frequency variants	0.5× lcWGS may be a practical depth for imputation-based applications
SNP-array imputation	25 K SNP-array data before and after imputation	Marker density increased from 18 to 180 to 1850–18,500 SNPs per 1 Mb window in representative regions	Imputation improved marker density and GWAS signal resolution

## Data Availability

None of the data was deposited in an official repository. The data are available upon request from the corresponding author.
